# Gender Dependency in Streoselective Pharmacokinetics of Tramadol and Its Phase I Metabolites in Relation to CYP2D6 Phenotype in Iranian Population

**Published:** 2018

**Authors:** Yalda Hosseinzadeh Ardakani, Hoda Lavasani, Mohammad-Reza Rouini

**Affiliations:** *Biopharmaceutics and Pharmacokinetics Division, Department of Pharmaceutics, Faculty of Pharmacy, Tehran University of Medical Sciences, Tehran, Iran.*

**Keywords:** Tramadol, Gender dependency Enantiomers, CYP2D6, Pharmacokinetics

## Abstract

The stereoselective pharmacokinetic of Tramadol (T) and its main metabolites concerning the influence of CYP2D6 phenotype and gender on the phase I metabolism of this compound was studied after administration of 100 mg single oral dose of racemic T to 24 male and female subjects. The pharmacokinetic parameters were estimated from plasma concentrations of the analytes enantiomers. The metabolic ratio of T enantiomers was used for CYP2D6 phenotype determination. The plasma concentrations of both tramadol enantiomers were considerably higher in Poor metabolizers (PM) than in extensive metabolizers (EM), resulting in 43% and 37% increase in AUC values of (+)-T and (-)-T respectively. The plasma concentrations of the (+)- and (-)-M1 enantiomers in EMs were significantly higher than the respective concentrations in PMs. The *N*-demethylation pathway was indirectly affected by CYP2D6 phenotypic differences. The plasma concentration of both enantiomers of M2 in PMs was higher than Ems. Although the concentration profiles and most of the calculated pharmacokinetic parameters of T and its main metabolites appears to be different in EMs and PMs, only the stereoselectivity of M1 enantiomers was significantly different in relation to CYP2D6 subgroups. No significant gender-related difference in the pharmacokinetics of T and its metabolites was observed.

## Introduction

Tramadol hydrochloride (T), a synthetic analog of codeine, contains two chiral centers with four stereoisomers. Trans-T, a racemic mixture of 1R, 2R-T ((+)–T), and 1S, 2S-T ((–)-T), produces analgesia by synergistic action with different binding properties of its enantiomers for various receptors (1). The analgesic activity of T was attributed to inhibition of serotonin and norepinephrine reuptake in addition to activation of μ-opioid receptors. In fact, the (+)-T preferentially inhibits serotonin reuptake, whereas (–)-T mainly inhibits norepinephrine reuptake and enhances norepinephrine release (1, 2). In addition to stereoselectivity in its pharmacodynamics, tramadol also exhibits stereoselective pharmacokinetics in humans mainly due to the stereoselective metabolism (3). Although the drug undergoes significant metabolism resulting in at least 23 metabolites in human urine (4), the major metabolic pathways are *O*-demethylation to *O*-desmethyl tramadol (M1) by the polymorphic isozyme cytochrome P450 2D6 (CYP2D6), and *N*-demethylation to *N*-desmethyl tramadol (M2) by cytochrome P450 2B6 (CYP2B6) and cytochrome P450 3A4 (CYP3A4) (1-4). These primary metabolites may be further metabolized to three additional secondary metabolites namely, *N, N*-Di dimethyl tramadol (M3), *N,N,O*-tri desmethyl tramadol (M4) and *N,O*-desmethyl tramadol (M5). The *O*-demethylated metabolites are then further conjugated with glucuronic acid and sulfate before excretion in urine (5).

Similar to the parent drug, the analgesic effects of M1, the only pharmacologically active metabolite, is stereoselective, then the (+)-M1 possesses a significant affinity for the μ-opioid receptors, whereas (–)-M1 inhibits monoamine reuptake. In fact, (+)-M1 has been demonstrated to have an affinity to μ-opioid receptors that are approximately 200 times greater than the parent compound (6). Thus, the (+)-enantiomer of M1 is responsible for opioid receptor mediated analgesia after administration of racemic tramadol and therefore the activity of CYP2D6, the most highly polymorphic isoenzyme of the cytochrome P450 system is important for the analgesic effects ascribed by this drug (7).

CYP2D6 has one of the best-characterized genetic factors known to affect drug metabolism in humans. The expression of this enzyme is deficient in approximately 7–10% of Caucasians (8).

The stereoselective disposition of tramadol and its active metabolite have been described (9-12). The pharmacokinetics of tramadol and M1 enantiomers in relation to CYP2D6 phenotype were reported in several ethnic groups (13-18). Most of the previous studies indicate that the pharmacokinetics and pharmacodynamics of tramadol and its main metabolites are both stereoselective and complex with a wide inter-individual variability. This may be due to the combined influence of factors such as CYP2D6 polymorphism or other factors like gender, age, and smoking status. 

Up to now, the gender dependency of tramadol pharmacokinetics and its main metabolites have fairly been investigated, especially in different phenotypic groups (11, 12 and 19). Hui-chen showed that there is a gender-related difference in the stereoselective pharmacokinetics of T in a human while the stereoselectivity in the pharmacokinetics of M1 was similar in both genders (11). Whereas, Quetglas demonstrated no pharmacokinetic differences of T, M1, and M2 enantiomers in Spanish male and female volunteers (12). Our previous study revealed no significant gender difference between the pharmacokinetic parameters of T and M1 metabolite, after non-stereoselective analysis. Although a trend of gender difference was observed for the M2 metabolite, the difference was not statistically significant; possibly, due to the wide inter-individual variability (19). The purpose of current study was to determine the stereoselective pharmacokinetic of tramadol and its metabolites concerning the influence of CYP2D6 phenotype and gender on the phase I metabolism of tramadol precisely. 

## Experimental

*Chemicals and Reagents*

The racemic forms and enantiomers of trans-tramadol, O-desmethyl tramadol (M1) and N-desmethyl tramadol (M2), as hydrochloride salts, were generously supplied by Grünenthal (Achen, Germany). Fluconazole (purity > 99%) (Internal standard, IS) was provided by Pars-Daru Co. (Tehran, Iran). HPLC-grade acetonitrile and analytical grade ethyl acetate, phosphoric acid (85%), triethylamine and diammonium hydrogen phosphate were supplied by Merck (Darmstadt, Germany). Water used in all experiments was of Direct-Q® quality (Millipore, France).

*Participants and study design*

Twenty-four healthy Iranian volunteers (12 male and 12 female) met the entry requirements and completed the study. The mean demographic data of age, height, and weight of volunteers are shown in Table 1.

The subjects were informed about the purpose of the study, gave their consent to participate, and received financial compensation. The protocol was approved by the Ethics Committee of Tehran University of Medical Sciences. Each participant underwent a general physical examination, routine laboratory tests, and urinalysis. The subjects were not allowed to take any other medication for 2 weeks before and throughout the study. Each subject fasted for 12 h before administration of two 50-mg Tradolan tablets (Lannach, Austria) with 200 mL of water and continued to fast for 3 h after administration. Standard breakfast and lunch were served 3 h and 6 h after dosing, respectively. The volunteers remained under close medical supervision until 10 h after the collection of the last blood samples.

*Sample collection*

The blood samples (3 mL) were collected in heparinized glass tubes before (time 0) and 0.5, 1, 1.5, 2, 2.5, 3.5, 4.5, 6, 8, 10 and 24 h after drug administration. Plasma was harvested after separation from blood cells by centrifugation and stored at -80 °C until analysis.

*Analytical method*

The concentrations of the enantiomers of tramadol and its metabolites were determined using a stereoselective high-performance liquid chromatography (HPLC) recently developed in our laboratory (20) Briefly, 500 µL plasma sample was transferred into a 15-mL glass tube and 50 μL of each IS solution and NaOH (2 M) were added. The samples were shaken with 6 mL of ethyl acetate for 15 min. The organic layer was separated after centrifugation (2 min), evaporated under a gentle stream of air and reconstituted in 120 μL of HCl (5 mM). A 100 μL aliquot of the sample was then injected into the HPLC system. The chromatographic experiments were performed in a Knauer high-performance liquid chromatography (Berlin, Germany), equipped with a low-pressure gradient HPLC pump with a fluorescence detector and a Rheodyne injector with a 100-µL loop. A complete resolution of tramadol enantiomers and its main phase I metabolites was performed by isocratic separation at room temperature (22 °C) using an AGP column with a mobile phase of 30 mM diammonium hydrogen phosphate buffer-acetonitrile-triethylamine (98.9:1:0.1, v/v), adjusted to pH 7 by phosphoric acid, and a flow rate of 0.5 mL/min. The fluorescence of analytes was detected at excitation and emission wavelengths of 200 and 301 nm. The calibration curves were linear (r2 > 0.993) in the concentration range of 2–200, 2.5–100, and 2.5–75 ng/mL for tramadol, M1, and M2 enantiomers, respectively. Precision and accuracy studies in plasma showed an acceptable R.S.D. values (≤ 14.2%) and accuracy (82.5-105%) for both between- and within-day studies. The lower limit of quantitation was 2 ng/mL for tramadol enantiomers and 2.5 ng/mL for M1 or M2 enantiomers. Mean recoveries of enantiomers from plasma samples were > 81% for all analytes (20).

*CYP CYP2D6 Genotyping*

The DNA from peripheral leukocytes was isolated by use of the SinaClon genomic DNA purification kit from CinnaGen (Iran), according to the manufacturer’s guidelines. Polymerase chain reaction with appropriate specific primers for the active CYP2D6 gene embracing the region with the four selected single nucleotide polymorphisms is the first step. The real-time analysis was executed on the ABI PRISM 7700 sequence detection system.

*Determination of CYP2D6 phenotype*

The two metabolic ratios for CYP2D6 phenotype determination with tramadol as probe were calculated using the area under the concentration versus time curves from zero to the last measured time point for enantiomers of tramadol and M1 (O-demethylated metabolite) as follows:

MR1=AUC0-24of+-tramadolAUC0-24 of +-M1

MR1=AUC0-24of--M1AUC0-24 of +-M1

As described by García Quetglas *et al.* (18), the logarithmic transform of MR1 and MR2 were used to determine the anti-mods by probit analysis. Probit transformations of the data were conducted by plotting the corresponding ratios of the AUC _(0–24)_ values against their corresponding percent areas under the normal probability curve (18).

The concordance between each of the two empirical metabolic ratios was assessed by calculating the sensitivity, specificity, positive predictive, and negative predictive values.

*Pharmacokinetic analysis*

The pharmacokinetics of tramadol and its metabolites were determined by noncompartmental analysis. Maximum plasma concentrations (Cmax) and their corresponding times (Tmax) were recorded as observed. Elimination rate constant (Ke) was estimated as the absolute value of the slope of the least-square linear regression of the terminal phase of the logarithmic plasma concentration–time curve. The plasma terminal half-life (T_1/2_) was calculated as 0.693/Ke. The Area under the plasma concentration–time curves from time zero to the time of last quantifiable concentration (AUC0–t) was calculated using the linear trapezoidal method. The Area under the plasma concentration–time curves from time zero to the infinite time (AUC0–∞) was calculated as the sum of corresponding AUC0–t and Ct/Ke values. Plasma oral clearance (CL/F) was calculated as Dose/AUC0–∞. The apparent volume of distribution (Vd/F) was determined using the equation Vd/F = (Dose/AUC0–∞)/Ke. Stereoselectivity was defined as the ratio of (+)/(-) enantiomers of analytes.

*Statistical analysis*

The differences between the pharmacokinetic parameters of the enantiomers of tramadol, M1 and M2 were analyzed using a two-tailed paired Student’s *t*-test. An unpaired *t*-test (two-tailed) was used to compare the differences in the pharmacokinetic parameters obtained for each enantiomer of all analytes between the poor and extensive metabolizer groups as well as the difference between two genders, except Tmax, with which a nonparametric Wilcoxon two-sample test was used. In all cases, a *p*-value of < 0.05 was considered to be significant. All results are expressed as mean ± SD.

## Results

**Figure 1:**
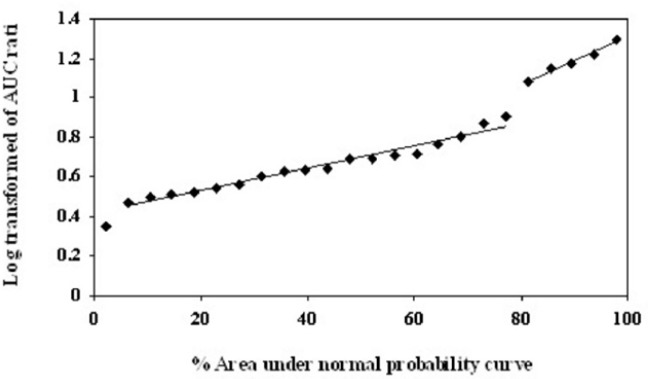
Probit analysis for the Log transformed of T/M1 ratio of the area under the concentration–time curves in 24 volunteers after 100 mg tramadol oral dose administration. The Y axis denotes the percent area under the normal probability curve for each data point. The X axis represents the AUC ratio values

The logarithmic transform of MR1 and the normal MR2 values are shown in Table 2. As shown in Figure 1, probit transformation of the two empirical ratios resulted in a nonlinear plot, with five data points deviated from the linear plot, and an anti-mod of approximately 1.0 and 2.0 for the logarithmic transform of MR1 and normal MR2 respectively. In all volunteers, both MR1 and MR2 values were clearly separated in EMs and PMs. The logarithmic transform of MR1 ranged from 0.349 to 1.4 (median of 0.689) and from 0.715 to 2.768 (median of 1.318) in MR2. In addition, the concordance between the two metabolic ratios was determined by calculation of the sensitivity, specificity, positive predictive value and negative predictive values. The sensitivity and specificity of the proposed phenotype test were 100%, with no misclassified subjects.

**Table 1:** Mean demographic data for subjects (n = 24).

	**Males**			**Females**		
	**Age (year)**	**Weight (kg)**	**Height (cm)**	**Age (year)**	**Weight (kg)**	**Height (cm)**
Mean	27.8	73.9	173.4	35.3	69.9	160.1
SD	7.0	10.2	4.4	6.3	10.3	10.4
Min	22	60	165	23	55	150
Max	42	80	180	42	85	165

**Table 2:** The phenotype of CYP2D6 from 24 subjects

**Subject**	**MR**_1_			**MR**_2_			**phenotype**
	**(+)-T** *	**(+)-M1** *	**Log**_10 _ **(T/M1)**	**(-)-M1** *	**(+)-M1** *	**((-)-M1/(+)-M1)**	
1	1255.3	217.5	0.761	256.9	217.5	1.181	EM
2	1525.7	291.5	0.719	432.9	291.5	1.485	EM
3	2019.4	122.3	1.218	268.4	122.3	2.194	PM
4	1950.0	99.1	1.294	213.9	99.1	2.159	PM
5	1218.5	336.2	0.559	313.8	336.2	0.933	EM
6	1480.7	223.2	0.821	305.5	223.2	1.368	EM
7	1442.6	226.8	0.803	326.5	226.8	1.440	EM
8	1241.1	422.3	0.468	352.1	422.3	0.834	EM
9	478.2	146.5	0.514	178.9	146.5	1.221	EM
10	989.5	442.5	0.349	473.7	442.5	1.070	EM
11	1602.1	204.1	0.895	199.9	204.1	0.979	EM
12	1073.7	342.7	0.496	354.4	342.7	1.034	EM
13	1240.6	242.0	0.710	344.3	242.0	1.423	EM
14	1438.2	119.5	1.081	255.1	119.5	2.135	PM
15	1003.5	235.8	0.629	359.5	235.8	1.525	EM
16	1247.7	310.6	0.604	356.8	310.6	1.149	EM
17	1012.5	288.0	0.546	379.5	288.0	1.318	EM
18	1999.0	134.3	1.173	371.6	134.3	2.768	PM
19	1541.8	110.2	1.146	256.6	110.2	2.330	PM
20	1093.8	329.4	0.521	235.5	329.4	0.715	EM
21	1241.5	280.0	0.647	313.1	280.0	1.118	EM
22	1262.8	255.6	0.690	382.7	255.6	1.485	EM
23	1353.5	315.7	0.632	354.6	315.7	1.123	EM
24	1017.5	208.6	0.688	274.9	208.6	1.318	EM

* : (ng h/mL).

**Table 3.** Pharmacokinetic parameters (mean ± SD) of the tramadol enantiomers and their main phase I metabolites after single oral administration (100 mg) of racemic Tramadol to 5 PMs and 19 EMs of CYP2D6

**Parameters**	**(+)-Enantiomer**		**(-)-Enantiomer**	
	**EMs**	**PMs**	**EMs**	**PMs**
Tramadol				
Cmax (ng/mL)	163.8 ± 27.7^a^, ^b^	203.8 ± 35.8^a^	150.9 ± 24.7^b^	186.2 ± 35.5
Tmax (h)	1.8 ± 0.4	1.8 ± 0.4	1.8 ± 0.5	1.8 ± 0.4
AUC_(0-t)_ (ng h/mL)	1225.19 ± 176.6^a^, ^b^	1789.7 ± 277.2^a^	1047.7 ± 150.6^b^	1450.6 ± 318.7
AUC_(0-∞)_ (ng h/mL)	1321.8 ± 202.2^a^, ^b^	1945.4 ± 365.4^a^	1109.1 ± 161.6^b^	1529.8 ± 311.7
Ke (1/h)	0.11 ± 0.03^a^	0.09 ± 0.02^a^	0.14 ± 0.07	0.11 ± 0.02
T_1/2 _(h)	6.7 ± 1.2^a^	7.7 ± 1.2	5.4 ± 1.3	7.2 ± 1.8
CL/F (mL/min)	635.3 ± 91.9^a^, ^b^	434.5 ± 72.0^a^	765.6 ± 108.1^b^	562.8 ± 111.5
Vd/F (l)	356.6 ± 148.3^b^	287.1 ± 44.3	360.7 ± 121.6	320.9 ± 51.4
M1				
Cmax (ng/mL)	32.8 ± 9.8^a^, ^b^	11.0 ± 4.5^a^	42.7 ± 9.3^b^	30.5 ± 6.9
Tmax (h)	2.4 ± 0.6	2.8 ± 1.0	2.3 ± 0.9	2.8 ± 1.2
AUC_(0-t)_ (ng h/mL)	299.1 ± 70.7^a^, ^b^	117.1 ± 13.2^a^	354.5 ± 50.5^b^	241.9 ± 24.2
AUC_(0-∞)_ (ng h/mL)	380.7 ± 85.4^a^, ^b^	157.2 ± 28.4^a^	404.5 ± 91.4^b^	273.8 ± 24.6
Ke (1/h)	0.10 ± 0.02^b^	0.06 ± 0.01^a^	0.11 ± 0.02	0.09 ± 0.01
T_1/2 _(h)	7.2 ± 2.0^b^	12.1 ± 2.0^a^	6.4 ± 0.9^b^	7.7 ± 0.8
M2				
Cmax (ng/mL)	18.5 ± 8.8^a^, ^b^	39.3 ± 12.5^a^	5.7 ± 2.8^b^	13.3 ± 5.9
Tmax (h)	2.6 ± 1.2^b^	4.5 ± 1.5	2.7 ± 0.9	3.5 ± 0.7
AUC_(0-t)_ (ng h/mL)	211.32 ± 106.1^a^,^ b^	502.2 ± 261.1^a^	78.5 ± 47.6^b^	183.1 ± 67.3
AUC_(0-∞)_ (ng h/mL)	277.6 ± 138.9 a, b	883.7 ± 644.5	105.6 ± 74.2	279.5 ± 33.5
Ke (1/h)	0.09 ± 0.04	0.07 ± 0.04	0.09 ± 0.02	0.07 ± 0.01
T_1/2 _(h)	9.3 ± 2.7	10.8 ± 4.1	8.2 ± 1.5	10.2 ± 0.9

a : *p* < 0.05 compared with the (-)-enantiomer in each subgroup.

b : *p* < 0.05 compared with the poor metabolizer group.

**Table 4:** (+)-Enantiomer/(-)-enantiomer ratios (mean ± SD) of pharmacokinetic parameters for tramadol and its main phase I metabolites after 100 mg single oral dose of racemic tramadol to 5 PMs and 19 EMs of CYP2D6

**Parameters**	**(+)-Enantiomer/(-)-enantiomer**	
	**EMs**	**PMs**
Tramadol		
Cmax	1.085 ± 0.040	1.098 ± 0.049
Tmax	0.983 ± 0.065	1.000 ± 0.00
AUC _(0-t)_	1.171 ± 0.066	1.254 ± 0.178
AUC _(0-∞)_	1.238 ± 0.068	1.354 ± 0.181
T_1/2_	1.157 ± 0.163	1.156 ± 0.162
CL/F	0.833 ± 0.046	0.783 ± 0.113
Vd/F	0.960 ± 0.109	0.900 ± 0.102
M1		
Cmax	0.769 ± 0.180**	0.362 ± 0.118
Tmax	1.139 ± 0.269	1.100 ± 0.476
AUC _(0-t)_	0.953 ± 0.217**	0.454 ± 0.021
AUC _(0-∞)_	1.119 ± 0.472*	0.529 ± 0.019
T_1/2_	1.459 ± 0.867**	1.505 ± 0.257
M2		
Cmax	4.385 ± 2.089	3.146 ± 0.688
Tmax	0.981 ± 0.212	1.290 ± 0.362
AUC _(0-t)_	4.487 ± 1.377	3.410 ± 1.261
AUC _(0-∞)_	4.926 ± 1.439	3.144 ± 2.082
T_1/2_	1.004 ± 0.364	1.095 ± 0.412

* *p* < 0.05,

** *p* < 0.01 compared with the poor metabolizer group.

**Table 5:** Pharmacokinetic parameters (mean ± SD) of the enantiomers of T and its main phase I metabolites in both genders with respect to CYP2D6 phenotype

**Parameters**	**(+)-Enantiomers**					**(-)-Enantiomers**			
	**EMs**		**PMs**			**EMs**		**PMs**	
	**Male**	**Female**	**Male**	**Female**		**Male**	**Female**	**Male**	**Female**
Tramadol									
Cmax (ng/mL)	183.4 ± 41.5	152.8 ± 51.9	214.9 ± 38.1	187.1 ± 35.9		170.0 ± 41.0	136.3 ± 44.4	194.7 ± 36.2	135.3 ± 11.2
Tmax (h)	1.6 ± 0.5	1.9 ± 0.4	1.7 ± 0.3	2.0 ± 0.7		1.6 ± 0.5	2.0 ± 0.4	1.7 ± 0.3	2.0 ± 0.7
AUC _(0-t)_*	1098.8 ± 302.1	1147.1 ± 390.7	1659.7 ± 298.4	1984.7 ± 49.0		941.8 ± 250.9	974.2 ± 326.3	1369.0 ± 306.8	1184.7 ± 140.0
AUC _(0-∞)_*	1328.8 ± 252.1	1343.1 ± 154.6	1864.6 ± 401.4	2103.6 ± 119.1		1089.4 ± 164.0	1046.5 ± 130.7	1471.7 ± 348.0	1285.0 ± 121.4
Ke (1/h)	0.10 ± 0.03	0.11 ± 0.02	0.10 ± 0.02	0.08 ± 0.01		0.12 ± 0.02	0.10 ± 0.01	0.12 ± 0.02	0.10 ± 0.02
T_1/2 _(h)	7.0 ± 1.3	6.4 ± 1.0	7.3 ± 1.2	8.3 ± 1.4		6.0 ± 0.8	6.6 ± 0.3	6.1 ± 0.9	7.0 ± 1.6
CL/F (mL/min)	644.3 ± 107.5	627.4 ± 75.7	459.6 ± 88.1	396.8 ± 22.5		779.7 ± 114.9	804.4 ± 98.0	586.3 ± 127.9	651.4 ± 61.6
Vd/F (l)	391.4 ± 94.1	352.2 ± 96.4	287.6 ± 42.6	286.5 ± 65.0		384.6 ± 90.2	463.5 ± 64.7	304.9 ± 54.6	397.1 ± 125.6
M1									
Cmax (ng/mL)	36.5 ± 12.9	35.2 ± 8.2	11.6 ± 5.3	10.1 ± 4.8	47.7 ± 14.4		47.7 ± 16.9	33.6 ± 3.7	27.2 ± 11.4
Tmax (h)	2.5 ± 1.4	2.4 ± 0.8	2.8 ± 1.2	2.8 ± 1.1	2.1 ± 1.1		2.4 ± 0.6	2.8 ± 1.5	2.8 ± 1.1
AUC _(0-t)_*	292.7 ± 98.2	310.6 ± 24.1	121.3 ± 12.1	110.7 ± 16.5	333.2 ± 90.6		325.0 ± 72.4	294.5 ± 66.9	263.8 ± 70.6
AUC _(0-∞)_*	381.5 ± 80.6	362.4 ± 55.2	167.5 ± 24.1	141.6 ± 21.4	383.6 ± 93.7		401.1 ± 48.1	358.0 ± 123.2	289.3 ± 59.5
Ke (1/h)	0.10 ± 0.02	0.09 ± 0.03	0.06 ± 0.01	0.07 ± 0.01	0.11 ± 0.01		0.09 ± 0.04	0.08 ± 0.02	0.08 ± 0.03
T_1/2 _(h)	7.0 ± 1.8	7.6 ± 2.6	12.6 ± 2.4	11.3 ± 1.1	6.3 ± 0.8		6.7 ± 1.2	8.6 ± 2.1	8.3 ± 1.3
M2									
Cmax (ng/mL)	18.6 ± 6.3	19.9 ± 14.5	36.7 ± 16.5	43.3 ± 5.5	6.3 ± 3.3		6.3 ± 4.7	13.9 ± 7.9	12.3 ± 3.7
Tmax (h)	2.4 ± 1.6	2.6 ± 0.8	4.3 ± 1.8	4.8 ± 1.8	3.0 ± 1.2		2.2 ± 0.3	3.5 ± 0.0	3.5 ± 1.4
AUC _(0-t)_*	234.8 ± 112.3	208.6 ± 114.4	368.1 ± 181.3	587.6 ± 262.5	80.9 ± 35.6		69.6 ± 32.1	215.9 ± 21.4	180.3 ± 94.0
AUC _(0-∞)_*	308.2 ± 157.3	283.1 ± 115.0	474.7 ± 248.2	834.7 ± 384.0	157.9 ± 46.8		145.5 ± 26.8	279.5 ± 33.5	264.9 ± 98.1
Ke (1/h)	0.06 ± 0.01	0.09 ± 0.03	0.07 ± 0.01	0.06 ± 0.02	0.09 ± 0.03		0.09 ± 0.02	0.06 ± 0.01	0.07 ± 0.01
T_1/2 _(h)	11.2 ± 1.5	8.3 ± 2.5	9.9 ± 1.2	12.0 ± 3.9	8.0 ± 1.7		8.3 ± 1.9	10.8 ± 0.5	9.6 ± 0.9

* *p* < 0.05

**Table 6:** (+)-Enantiomer/(-)-enantiomer ratios (mean ± SD) of pharmacokinetic parameters of tramadol and its main phase I metabolites in both genders with respect to CYP2D6 phenotype

**Parameters**	**(+)-Enantiomer/(-)-enantiomer**			
	**EMs**		**PMs**	
	**Male**	**Female**	**Male**	**Female**
Tramadol				
Cmax	1.083 ± 0.048	1.103 ± 0.052	1.106 ± 0.052	1.085 ± 0.062
Tmax	1.000 ± 0.000	0.969 ± 0.088	1.000 ± 0.000	1.000 ± 0.000
AUC _(0-t)_	1.168 ± 0.040	1.177 ± 0.085	1.222 ± 0.111	1.302 ± 0.308
AUC _(0-∞)_	1.216 ± 0.073	1.201 ± 0.087	1.274 ± 0.101	1.340 ± 0.362
T_1/2_	1.242 ± 0.142	1.103 ± 0.044	1.207 ± 0.099	1.095 ± 0.212
CL/F	0.825 ± 0.048	0.836 ± 0.056	0.788 ± 0.061	0.775 ± 0.209
Vd/F	1.020 ± 0.082*	0.921 ± 0.059	0.950 ± 0.097	0.826 ± 0.065
M1				
Cmax	0.761 ± 0.168	0.771 ± 0.171	0.349 ± 0.161	0.383 ± 0.047
Tmax	1.231 ± 0.249	1.000 ± 0.214	1.059 ± 0.315	1.161 ± 0.833
AUC _(0-t)_	0.881 ± 0.170	0.901 ± 0.240	0.420 ± 0.054	0.459 ± 0.005
AUC _(0-∞)_	1.051 ± 0.370	1.169 ± 0.594	0.487 ± 0.087	0.484 ± 0.040
T_1/2_	1.456 ± 1.003	1.011 ± 0.254	1.493 ± 0.274	1.273 ± 0.098
M2				
Cmax	4.511 ± 2.729	3.993 ± 1.202	3.603 ± 0.633	4.511 ± 2.729
Tmax	0.881 ± 0.341	1.000 ± 0.000	1.367 ± 0.047	0.881 ± 0.344
AUC _(0-t)_	5.240 ± 2.930	4.338 ± 0.408	4.147 ± 0.846	5.240 ± 2.903
AUC _(0-∞)_	5.109 ± 1.930	4.561 ± 0.828	4.639 ± 1.032	5.144 ± 1.789
T_1/2_	1.196 ± 0.067	1.139 ± 0.076	1.178 ± 0.081	1.346 ± 0.181

* *p* < 0.05 compared with female.

**Table 7:** Percent of metabolic ratios (mean ± SD) of tramadol and its main phase I metabolites in the case of Cmax and AUC in both genders with respect to CYP2D6 phenotype

**Parameters**	**(+)-Enantiomers**				**(-)-Enantiomers**			
	**Cmax**		**AUC **_(0-t)_		**Cmax**		**AUC **_(0-t)_	
	**Male**	**Female**	**Male**	**Female**	**Male**	**Female**	**Male**	**Female**
EMs								
M1/T	18.8 ± 6.6	1999.6 ± 1.8	28.0 ± 10.4	25.0 ± 4.2	25.8 ± 6.8	25.2 ± 4.0	32.5 ± 9.5	28.4 ± 6.3
M2/T	10.5 ± 3.8	9.8 ± 4.9	17.6 ± 7.1	15.6 ± 6.4	4.1 ± 2.0	5.5 ± 5.1	6.7 ± 5.3	5.9 ± 4.2
PMs								
M1/T	5.5 ± 2.7	5.2 ± 1.6	7.4 ± 0.8	5.6 ± 0.7	17.9 ± 5.4	20.5 ± 10.1	21.5 ± 1.0	22.8 ± 8.7
M2/T	16.3 ± 7.3	23.9 ± 7.5	22.2 ± 11.4	29.7 ± 10.2	9.4 ± 0.5	9.0 ± 2.0	15.5 ± 2.9	13.0 ± 5.5

**Figure 2:**
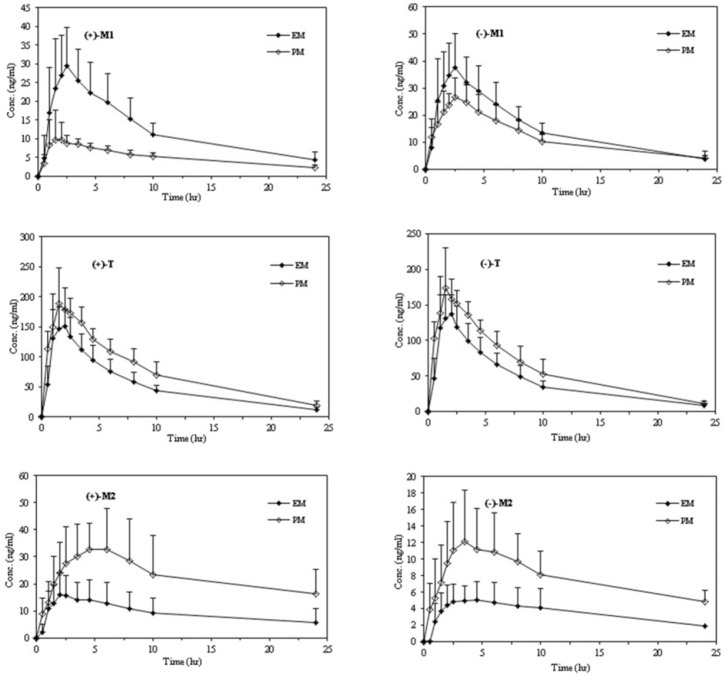
Mean plasma concentration–time profiles of enantiomers of T, M1, and M2 after oral administration of racemic tramadol (100 mg) in extensive and poor CYP2D6

**Figure 3:**
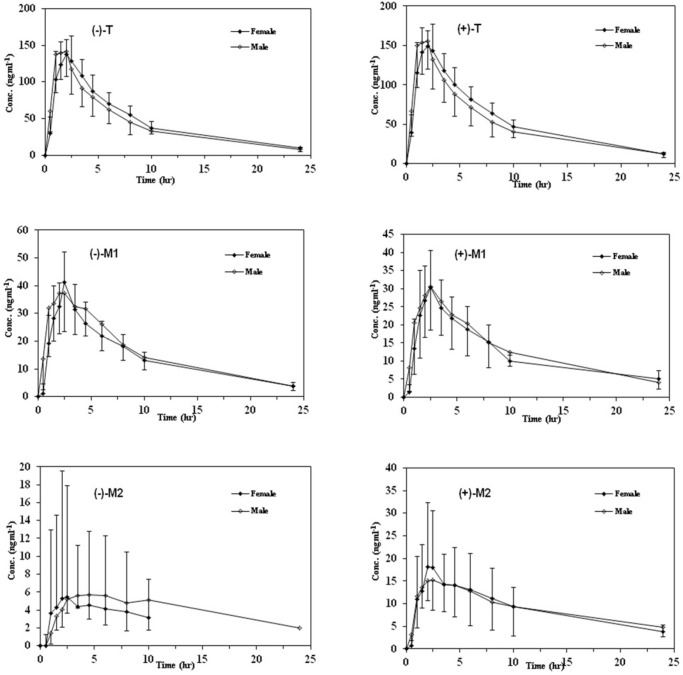
Mean plasma concentration–time profiles of enantiomers of T, M1, and M2 after oral administration of racemic tramadol (100 mg) in male and female EM subjects

**Figure 4:**
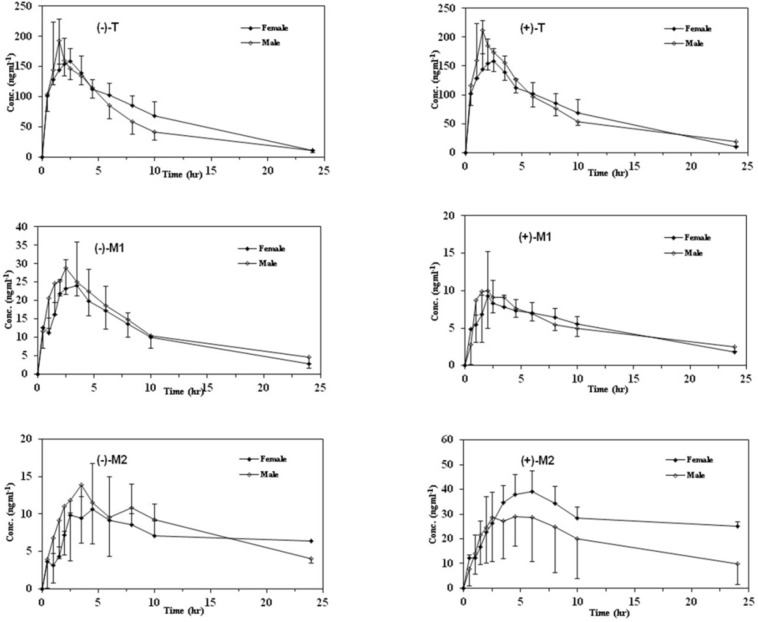
Mean plasma concentration–time profiles of enantiomers of T, M1, and M2 after oral administration of racemic tramadol (100 mg) in male and female PM subjects

Three of the EMs and one of the PMs experienced moderate adverse events consisting of dizziness, nausea, and tiredness 45-60 min after administration of 100 mg racemic tramadol. None of the adverse events was serious; therefore, all participants completed the study.

The concentrations for both enantiomers of T and M1 were detectable at all sampling times in all volunteers regardless of phenotype. (+)-M2 was not detected in two EMs (from male subjects) at two latest sampling times whereas (-)-M2 concentrations were a below limit of quantification in 8 of 19 EMs (5 of males and 3 of females) almost at all measurements and in 4 of 19 EMs at two latest sampling times. Due to the lack of data points, most pharmacokinetic parameters of (-)-M2 were calculated based on less than nineteen EMs. Both enantiomers of M2 were detectable in all PMs Most probably due to accumulation of N-demethylated metabolite (M2) in the subjects with a phenotype deficient on CYP2D6 activity in comparison with extensive metabolizers (18, 21).

Henceforth, the difference in the concentration–time profiles and pharmacokinetic parameters of enantiomers of T and its main metabolites are evaluated and presented in three steps. First, the pharmacokinetic differences of enantiomers (each analyte) between the EMs and PMs are investigated. Second, the pharmacokinetic differences of both enantiomers (each analyte) are evaluated in each phenotype, and finally, statistical differences in the (+)/(–)-enantiomeric ratios (the stereoselectivity) of the calculated parameters of each analyte are evaluated between EMs and PMs.

*Tramadol*

The mean plasma concentration–time courses of tramadol enantiomers in relation to the CYP2D6 phenotype are depicted in Figure2 and a summary of the major pharmacokinetic parameters including the statistical inferences of the differences between the pharmacokinetic parameters of the tramadol enentiomers and each enantiomer of all analytes between the EMs and PMs are presented in Table 3.

After oral administration of racemic tramadol, significant differences between the two phenotypes in most pharmacokinetic parameters were observed. The concentrations of both enantiomers of parent drug were found to be higher in the PMs than in the EMs at most of the sampling times (Figure 2 and Table 3).

Even though the maximum plasma concentrations occurred nearly at the same time for (+)- and (-)-T in two subgroups, the corresponding Cmax and AUC values were considerably higher in PMs in comparison to EMs. Poor metabolizers had approximately 23% increase in Cmax and 43% in AUC of (+)-T, whereas the corresponding increase in same parameters of (-)-T were 22% and 37% respectively. On the other hand, the significant lower clearance values (CL/F) of both enantiomers of T in PMs than in EMs may result in a slower elimination of T in this group and therefore a longer elimination of half-life is expected. A trend towards higher Vd/F values for EMs than PMs, especially for (+)-enantiomer was observed, however, the differences did not reach statistical significance when two phenotypic subgroups were compared. 

The statistical differences between the pharmacokinetic parameters of the tramadol enantiomers in each phenotype subgroups were also investigated. There were statistically significant differences between most of the pharmacokinetic parameters of tramadol enantiomers in each phenotype subgroup (Table 3). 

No statistical difference in the (+)/(–)-enantiomeric ratios of calculated parameters were observed for the parent compound between two phenotypes, suggesting that the stereoselectivity of the racemic tramadol disposition is less influenced by the CYP2D6 phenotype.

*O-desmethyl tramadol (M1)*

The mean plasma concentration–time profiles of the enantiomers of M1 and the pharmacokinetic parameters in relation to the CYP2D6 phenotype are shown in Figure 2 and Table 3. 

The enantiomers of M1 were detectable in plasma from the first sampling time (30 min) in most volunteers. The Tmax values of both enantiomers in PMs were higher than EMs with no statistical difference (2.8 h versus 2.4 h). The plasma concentrations of the both (+)- and (-)- enantiomers reflected in the Cmax and AUC values, were considerably higher in EMs than PMs (*p *< 0.001). 

Besides, the statistical inferences of the differences between the pharmacokinetic parameters of the enantiomers of M1 in each phenotype subgroups were studied. The times to achieve the maximum plasma concentration were equal for both enantiomers of M1 in each phenotype. However, significant differences were found in terms of Cmax and AUC values between (+)- and (-)-M1 in each phenotypic subgroups. Furthermore, the difference between the Cmax and AUC values of (+)- and (-)-M1 in PMs is much higher than EMs (*p*-value of 0.0004 versus 0.0186 in the case of Cmax and 0.0003 versus 0.0048 in the case of AUC_ (0-t)_). PMs had also statistically various elimination rate constant (ke) and half-life (T_1/2_) values between (+) and (-)-M1, while no such a difference was observed in EMs. 

In contrast to the parent drug, there were significant differences between the enantiomeric ratios of the M1 metabolite in the Cmax and AUC values showing that stereoselectivity of the M1 disposition is influenced by the CYP2D6 phenotype (Table 4). 

*N-desmethyl tramadol (M2)*

The mean plasma concentration–time profiles of the M2 enantiomers and the pharmacokinetic parameters in relation to the CYP2D6 phenotype during the study are shown in Figure 2 and Table 3. N-desmethyl tramadol (M2) was not immediately quantifiable in plasma after tramadol administration, with a lag time of 0.5 to 2 h. 

Although maximum plasma concentrations of both enantiomers of M2 occurred nearly later in PMs than EMs, the statistical difference was observed just for the (+)-M2 between 2D6 subgroups. As mentioned previously, because of a metabolic switch in favor of enhanced N-demethylation of T in the presence of low CYP2D6 activity in the PMs, the plasma concentrations of both enantiomers of M2 (reflected in Cmax and AUC values) were significantly higher in PMs than Ems (18, 21).

Although significant differences in the Cmax and AUC values were observed between (+)- and (-)- enantiomers of M2 in each phenotype, the difference was considerably higher between EMs especially in Cmax values (*p*-value < 0.0001 for EMs and *p-*value < 0.01 for PMs) (Table 3). The largest difference in the Cmax values between the M2 enantiomers was approximately 9-fold in one of the EMs with a mean value of about 4.4 in this subgroup.

Among all analytes, the largest value of the (+)/(−) ratio was observed for M2 metabolite with no significant difference between two phenotypes especially due to lack of interference of CYP2D6 in the formation of this metabolite (Table 4). On the other hand, the stereoselectivity values of the pharmacokinetics of the generated metabolite (M2) were not significantly different between extensive and poor metabolizers. 

*Effects of gender on pharmacokinetics of tramadol and its main metabolites*

In order to provide information regarding the influence of gender on stereoselective pharmacokinetics of T and its main phase I metabolites with respect to CYP2D6 phenotype, mean plasma concentration–time profiles of the enantiomers of all analytes in both genders are depicted in Figures 3 and 4 in EMs and PMs and the corresponding pharmacokinetic variables are summarized in Table 5. 

Although the maximum plasma concentrations of both enantiomers of T in the plasma occurred relatively later in female than male subjects in both phenotypes, there was no significant difference in all calculated pharmacokinetic parameters between the male and female subjects (*p *> 0.05). In addition, the (+)/(–)–enantiomeric ratios of the pharmacokinetic parameters for T were not significantly different in males and in females in either EMs or PMs (Figure 3).

Mean plasma concentration–time curves of M1 and M2 enantiomers in both genders with respect to CYP2D6 phenotypes are shown in Figures 3 and 4 and the related pharmacokinetic parameters are reviewed in Table 5. The time to reach the maximum plasma concentration of enantiomers of metabolites took place approximately 0.5 h and 0.5-1 h after the Tmax of T for M1 and M2 in both cited subgroups respectively. Similar to the parent compound, there were no significant differences in all calculated parameters as well as the enantiomeric ratios of corresponding parameters between the genders in all mentioned subgroups (Tables 5 and 6). Besides, there were no significant gender differences in the metabolic ratios (metabolite/Tramadol) of each enantiomer of metabolites in both phenotypes in the case of Cmax and AUCs (Table 7). 

## Discussion

The biotransformation of (+)-T to (+)-M1 is exclusively mediated by the CYP2D6 isozyme. Laugesen suggested that although the other enzymes may also contribute to the generation of (-)-M1 metabolite from (-)-T, CYP2D6 is practically the only enzyme responsible for (+)-M1 formation (23). The importance of CYP2D6 activity due to the formation of (+)-M1 for the analgesic effect of tramadol using human experimental pain models has also been found by Poulsen and Stamer (7, 24). They reported a lower response rate to postoperative tramadol analgesia in PMs compared to EMs, as PMs could hardly form any (+)-M1 and much lower levels of (-)-M1 are also generated in this group in comparison with EMs. Therefore, there is an interest in providing information on the incidence of the poor metabolizer phenotype of CYP2D6 in the population and pharmacokinetics of tramadol and its phase I metabolites regarding the influence of these phenotype subgroups. Although Sparteine is one of the best probes with a clear-cut bimodal distribution for CYP2D6 phenotyping, there is an interest for new validated probes because of difficulties in obtaining the commercial formulations of this compound. 

Recently, tramadol was introduced as a probe by Pedersen (14), providing an alternative to sparteine for *in-vivo* phenotyping of CYP2D6. The authors suggested the 8-h urinary metabolic ratio of (−)-M1 to (+)-M1 with an anti-mode of 2.0 for separating EMs and PMs. However, the evaluation of the plasma concentration potential for CYP2D6 activity was not possible because of the lack of sensitive analytical method (14). Recently, García-Quetglas successfully separated the subgroups of CYP2D6 phenotype in healthy Spanish subjects using the ratio of area under the plasma concentration versus time curves of (+)-T to (+)-M1 after oral administration of racemic compound (18). Although Pedersen reported an unclear-cut bimodal distribution for MR2, García-Quetglas suggested that both MR1 and MR2 could successfully separate the subgroups of 2D6 phenotype after probit analysis of the experimental data in plasma with no misclassified subject (14, 18). They reported the anti-mode value of 1.0 and 5.0 for log transformed MR1 and normal MR2 respectively and classified the subjects as PMs (5 of 24 volunteers or 20%) and EMs (19 of 24 population or 80%) (18). Similar to the subsequent report, the probit transformation of the two empirical ratios of our subjects resulted in a nonlinear plot, with five data points deviated from the linear plot (20% of our population study), and an anti-mode of approximately 1.0 and 2.0 for logarithmic transformed MR1 and normal MR2 respectively (18). Although all volunteers were clearly separated in EMs and PMs with MR1 or MR2, the higher plasma concentration of (+)-M1 was observed in our population study, especially in PMs. This higher (+)-M1 concentration may result in the difference of MR2 anti-mode value between two studies. 

Except for (+)-M1 plasma concentrations in PMs, the mean pharmacokinetic parameters of tramadol enantiomers and (-)-M1 were comparable to the values published by Fliegert and García-Quetglas in EM and PM Populations (15, 18). The concentration–time profiles of (+)-M1 in PMs of our study were approximately two-fold higher in Cmax and AUCs than those reported by García-Quetglas (18). Unfortunately, the concentration–time profiles of (+)-M1 in PMs could not be compared with Fliegert (15); due to the lack of sensitive analytical method they were not able to calculate parameters. The mean pharmacokinetic parameters of M2 enantiomers in our study are comparable to the only comprehensive reported disposition of M2 enantiomers in relation to CYP2D6 phenotypes by García-Quetglas (18).

Similar to cited reports, the statistical inference of our findings show the differences of the pharmacokinetic parameters of both T enantiomers (except for Tmax) between EMs and PMs as well as differences between the pharmacokinetic parameters of the enantiomers of this compound in each phenotype subgroups. However, the stereoselectivity of tramadol disposition ((+)/(-) enantiomeric ratios) is less influenced by the CYP2D6 phenotype. 

The pharmacokinetic parameters of M1 enantiomers were also markedly different between the two phenotypes. The plasma concentrations of (+)- and (-)- M1 in EMs, especially reflected in the Cmax and AUC values, were significantly higher than the respective values in PMs. While both enantiomers demonstrated statistical differences between two phenotypes, the difference between the Cmax and AUC values of (+)-M1 is much higher than (-)-M1 between 2D6 subgroups (*p*-value of 0.0004 versus 0.0186 in the case of Cmax and *p*-value of 0.0001 versus 0.0087 in the case of AUC _(0-∞)_ for (+)- and (-)-enantiomers respectively). This finding is in agreement with García-Quetglas (18). Suggesting that (−)-M1 formation is less influenced by the CYP2D6 genetic polymorphism. 

In contrast to the parent drug, the stereoselectivity of the M1 disposition is influenced by the CYP2D6 phenotype, as EMs show about 2 fold higher (+)/(−)-Cmax and AUC ratio values than PMs (Table 4). 

As it was mentioned above, a metabolic switch in favor of enhanced N-demethylation of T has been suggested in the presence of low CYP2D6 activity in the PM phenotype population (18, 21). This metabolic switch may lead to an increase in the M2 concentration level in PMs. Despite the presence of more substrate (M2) available for M5 formation, no more M5 may be formed as a result of decreased O-demethylation of M2 by 2D6. Re-analysis of our previous non-stereoselective pharmacokinetic data according to phenotype subgroups confirmed the cited assumption. Assuming the O-demethylation as the rate limiting step, no more M5 metabolite was formed in PMs in spite of much higher concentration of M2 in this subgroup in comparison to EMs (the re-analysed data was not shown). (19) Although García-Quetglas (18) reported the influence of 2D6 phenotype on stereoselectivity of both metabolites of tramadol, no statistical difference in the stereoselectivity of M2 metabolite was shown in relation to 2D6 phenotypes in our study population. 

Nowadays there is a growing concern about gender differences in pain management. A considerable body of evidence indicates that there are gender differences in opioid antinociception in humans and experimental animals, with males generally displaying greater antinociceptive sensitivity than females especially for intermediate to low efficacy agonists (25). Similarly, Dai demonstrated pharmacodynamic related differences in genders resulting in less sensitivity of female mice to tramadol-induced antinociception than males (26). By contrast, Liu found a higher rate of O-demethylation of tramadol resulting in higher Cmax and AUC of the M1 metabolite in females than in males (22). In accordance with our previous non-stereoselective pharmacokinetic study (19), no statistical differences were found between two genders neither in all calculated stereoselective pharmacokinetic parameters nor in enantiomeric ratios even after separating EMs and PMs. This finding is in concordance with the results of the study of Quetglas showing no pharmacokinetic differences of T, M1, and M2 enantiomers obtained from Spanish male and female volunteers (12). 

## Conclusions

The phase I metabolism of tramadol appears to be dependent on the polymorphism of CYP2D6. Although the concentration profiles and most of the calculated pharmacokinetic parameters of T and its main metabolites appears to be different in EMs and PMs, only the stereoselectivity of the M1 enantiomers was different in relation to 2D6 subgroups. As the opioid effects of (+)-M1 plays an important role on the analgesic effect of tramadol and it was suggested that μ-opioid receptor plays a crucial role in tramadol-induced antinociception (26), the biotransformation of tramadol to (+)-M1 via CYP2D6 is assumed to have an important impact on the analgesic response. In spite of pharmacodynamics differences demonstrated in sexes, no pharmacokinetic differences were found in two genders.

## References

[1] Raffa RB, Friderichs E, Reimann W, Shank RP, Codd EE, J Vaught L (1992). Opioid and nonopioid components independently contribute to the mechanism of action of tramadol, an ‘atypical’ opioid analgesic. J. Pharmacol. Exp. Ther.

[2] Raffa RB, Friderichs E, Reimann W, Shank RP, Codd EE, Vaught JL, Jacoby HI, Selve N (1993). Complementary and synergistic antinociceptive interaction between the enantiomers of tramadol. J. Pharmacol. Exp. Ther.

[3] Paar WD, Frankus P, Dengler HJ (1992). The metabolism of tramadol by human liver microsomes. Clin. Investig.

[4] Wu WN, McKown LA, Liao S (2002). Metabolism of the analgesic drug ULTRAM (tramadol hydrochloride) in humans: API-MS and MS/MS characterization of metabolites. Xenobiotica.

[5] Lintz W, Erlaçin S, Frankus E, Uragg H (1981). Biotransformation of tramadol in man and animal (author’s trans). Arzneimittelforschung.

[6] Gillen C, Haurand M, Kobelt DJ, Wnendt S (2000). Affinity, potency and efficacy of tramadol and its metabolites at the cloned human mu-opioid receptor. Naunyn Schmiedebergs Arch. Pharmacol.

[7] Poulsen L, Arendt-Nielsen L, Brøsen K, Sindrup SH (1996). The hypoalgesic effect of tramadol in relation to CYP2D6. Clin. Pharmacol. Ther.

[8] Bertilsson L (1995). Geographical/interracial differences in polymorphic drug oxidation Current state of knowledge of cytochromes P450 (CYP) 2D6 and 2C19. Clin. Pharmacokinet.

[9] Liu HC, Liu TJ, Yang YY, Hou YN (2001). Pharmacokinetics of enantiomers of trans-tramadol and its active metabolite, trans-O-demethyltramadol, in human subjects. Acta Pharmacol. Sin.

[10] Campanero MA, García-Quetglas E, Sádaba B, Azanza JR (2004). Simultaneous stereoselective analysis of tramadol and its primary phase I metabolites in plasma by liquid chromatography Application to a pharmacokinetic study in humans. J. Chromatogr. A.

[11] Hui-Chen L, Yang Y, Na W, Ming D, Jian-Fang L, Hong-Yuan X (2004). Pharmacokinetics of the enantiomers of trans-tramadol and its active metabolite, trans-O-demethyltramadol, in healthy male and female chinese volunteers. Chirality.

[12] García Quetglas E, Azanza JR, Cardenas E, Sádaba B, Campanero MA (2007). Stereoselective pharmacokinetic analysis of tramadol and its main phase I metabolites in healthy subjects after intravenous and oral administration of racemic tramadol. Biopharm. Drug Dispos.

[13] Pedersen RS, Damkier P, Brosen K (2006). Enantioselective pharmacokinetics of tramadol in CYP2D6 extensive and poor metabolizers. Eur. J. Clin. Pharmacol.

[14] Pedersen RS, Damkier P, Brosen K (2005). Tramadol as a new probe for cytochrome P450 2D6 phenotyping: A population study. Clin. Pharmacol. Ther.

[15] Fliegert F, Kurth B, Göhler K (2005). The effects of tramadol on static and dynamic pupillometry in healthy subjects--the relationship between pharmacodynamics, pharmacokinetics, and CYP2D6 metabolizer status. Eur. J. Clin. Pharmacol.

[16] Slanar O, Nobilis M, Kvetina J, Idle JR, Perlík F (2006). CYP2D6 polymorphism, tramadol pharmacokinetics and pupillary response. Eur. J. Clin. Pharmacol.

[17] Enggaard TP, Poulsen L, Arendt-Nielsen L, Brøsen K, Ossig J, Sindrup SH (2006). The analgesic effect of tramadol after intravenous injection in healthy volunteers in relation to CYP2D6. Anesth. Analg.

[18] García-Quetglas E, Azanza JR, Sádaba B, Muñoz MJ, Gil I, Campanero MA (2007). Pharmacokinetics of tramadol enantiomers and their respective phase I metabolites in relation to CYP2D6 phenotype. Pharmacol. Res.

[19] Ardakani YH, Rouini MR (2007). Pharmacokinetics of tramadol and its three main metabolites in healthy male and female volunteers. Biopharm. Drug Dispos.

[20] Ardakani YH, Mehvar R, Foroumadi A, Rouini MR (2008). Enantioselective determination of tramadol and its main phase I metabolites in human plasma by high-performance liquid chromatography. J. Chromatogr. B.

[21] Abdel-Rahman SM, Leeder JS, Wilson JT (2002). Concordance between tramadol and dextromethorphan parent/metabolite ratios: the influence of CYP2D6 and non-CYP2D6 pathways on biotransformation. J. Clin. Pharmacol.

[22] Liu HC, Wang N, Yu Y, Hou YN (2003). Stereoselectivity in trans-tramadol metabolism and trans-O-demethyltramadol formation in rat liver microsomes. Acta Pharmacol. Sin.

[23] Laugesen S, Enggaard TP, Pedersen RS, Sindrup SH, Brosen K (2005). Paroxetine, a cytochrome P450 2D6 inhibitor, diminishes the stereoselective O-demethylation and reduces the hypoalgesic effect of tramadol. Clin. Pharmacol. Ther.

[24] Stamer UM, Lehnen K, Höthker F, Bayerer B, Wolf S, Hoeft A, Stuber F (2003). Impact of CYP2D6 genotype on postoperative tramadol analgesia. Pain.

[25] Craft RM, Tseng AH, McNiel DM, Furness MS, Rice KC (2001). Receptor-selective antagonism of opioid antinociception in female versus male rats. Behav. Pharmacol.

[26] Dai X, Brunson CD, Rockhold RW, Loh HH, Ho IK, Ma T (2008). Gender differences in the antinociceptive effect of tramadol, alone or in combination with gabapentin, in mice. J. Biomed. Sci.

[27] Tanaka E (1999). Gender-related differences in pharmacokinetics and clinical significance. J. Clin. Pharm. Ther.

